# Reliability and validity of ultrasound imaging of features of knee osteoarthritis in the community

**DOI:** 10.1186/1471-2474-12-70

**Published:** 2011-04-06

**Authors:** Ajay M Abraham, Iain Goff, Mark S Pearce, Roger M Francis, Fraser Birrell

**Affiliations:** 1 Northumbria Healthcare NHS Trust, Northumberland, UK; 2 Institute of Health and Society, Newcastle University, Newcastle, UK; 3 Newcastle upon Tyne Hospitals NHS Foundation Trust, Newcastle, UK; 4 Institute for Ageing and Health, Newcastle University, Newcastle, UK; 5 Musculoskeletal Research Group, Newcastle University, Newcastle, UK

**Keywords:** Musculoskeletal ultrasound, knee osteoarthritis, cartilage, bone, reliability, validity

## Abstract

**Background:**

Radiographs are the main outcome measure in epidemiological studies of osteoarthritis (OA). Ultrasound imaging has unique advantages in that it involves no ionising radiation, is easy to use and visualises soft tissue structures. Our objective was to measure the inter-rater reliability and validity of ultrasound imaging in the detection of features of knee OA.

**Methods:**

Eighteen participants from a community cohort, had both knees scanned by two trained musculoskeletal sonographers, up to six weeks apart. Inter-rater reliability for osteophytes, effusion size and cartilage thickness was calculated by estimating Kappa (κ) and Intraclass correlation coefficients (ICC), as appropriate. A measure of construct validity was determined by estimating κ between the two imaging modalities in the detection of osteophytes.

**Results:**

*Reliability: *κ for osteophyte presence was 0.77(right femur), 0.65(left femur) and 0.88 for both tibia. ICCs for effusion size were 0.70(right) and 0.85(left). Moderate to substantial agreement was found in cartilage thickness measurements. *Validity: *For osteophytes, κ was moderate to excellent at 0.52(right) and 0.75(left).

**Conclusion:**

Substantial to excellent agreement was found between ultrasound observers for the presence of osteophytes and measurement of effusion size; it was moderate to substantial for femoral cartilage thickness. Moderate to substantial agreement was observed between ultrasound and radiographs for osteophyte presence.

## Background

The epidemiological study of knee osteoarthritis (OA) has had many barriers; a major problem being the definition of knee OA. The most commonly used outcome measure in studies of OA has been radiological criteria, such as those described by Kellgren and Lawrence [1]. Radiographs have limitations, such as the need for low-level radiation exposure and the inability to view soft tissue structures and assess inflammation. Furthermore, it also has the disadvantage of obtaining only two dimensional images from one or more views. However, although imperfect, radiographs still remain the closest to a gold standard for epidemiological studies of knee OA [2].

Inflammation has been shown to be a consistent feature of OA and has also been found to contribute to its progression [3-5]. Demonstration of inflammation requires more sensitive modalities like ultrasound and magnetic resonance imaging (MRI); the inflammation can also then be quantified. A recent paper by Iagnocco et al demonstrated a high prevalence of ultrasound defined effusions (43%) in 82 patients with knee OA in Italy and also showed a high correlation between the total ultrasound score and the Lequesne index (a validated measure of severity of knee OA) as well as the patient's global assessment of knee pain, which provides some evidence for its concurrent validity [6]. Ultrasound has the advantage over MRI in that it is cheaper, convenient and easier to use, is dynamic and has no contra-indications to its use [7]. Ultrasound involves no radiation and can obtain views in multiple planes. It can also visualise soft tissue structures like the menisci [8] and cartilage [9,10], which are known to be involved in the pathophysiology and progression of OA [11,12]. Indeed, OA is now regarded as a failure of the joint as an organ, much like renal or cardiac failure [13] and it becomes imperative that we use an imaging outcome that is able to visualise the various structures within and around the joint, to ensure criterion validity of the outcome measure. It is therefore not surprising that organisations such as OMERACT and OARSI are in the process of developing research agendas on the use of ultrasound in OA [14].

Inter-rater reliability of ultrasound features of OA has been documented in several studies of hospital based participants [15-18]. A study of patients with knee pain due to various arthritides and referred for arthroscopy in Leeds, UK, demonstrated that the Kappa for inter-rater agreement for presence or absence of synovitis in the knees on a subset of 10 patients was 0.71; while weighted kappa for distinguishing the grade of synovitis was 0.65 [15]. Inter-rater reliability for ultrasound measurements of femoral condylar cartilage thickness demonstrated ICCs between 0.75 and 0.96 in a study of eight cadaveric knees [19]. There is a need for inter-rater reliability studies of ultrasound imaging, especially for further evidence of specific features of knee OA such as hyaline cartilage thickness and osteophytes.

There have been studies in secondary care that demonstrate the validity of ultrasound in OA and other arthritides. Ultrasound compared well with MRI in the assessment of femoral condylar cartilage, effusion and synovial thickening in a study of 58 patients with symptomatic knee OA [20]. Karim provided evidence for the validity of ultrasound in detecting synovitis in the knee, when compared with macroscopic arthroscopic findings, in 60 patients with various arthritides in Leeds [15]. The agreement between ultrasonographic and histological measurements of femoral cartilage thickness was demonstrated to be high, with ICCs of 0.73 to 0.88 [19]. This was a study which compared ultrasound with macroscopic anatomic findings from seven cadaveric knees. The validity of femoral cartilage thickness measured by ultrasound was also demonstrated by comparison of ultrasound derived cartilage thickness measurement with histology in a cohort of 18 patients with severe knee OA who underwent subsequent knee arthroplasty. There was excellent correlation between ultrasound and histology derived cartilage thickness, that was found to be statistically significant [21]. Comparison of femoral cartilage thickness between ultrasound and MRI in a Danish study has shown a Spearman correlation coefficient of 0.82, in 20 subjects with various arthritides, including OA [22].

Despite evidence for the validity of femoral cartilage thickness measurements using ultrasound, there is very little evidence for the validity of osteophyte detection with ultrasound, in patients with knee OA [14]. Nevertheless, ultrasound has been found to be superior to radiographs in the detection of osteophytes in hand OA [23]. Meenagh et al concluded in their review paper that osteophytes can and should be visualised in studies of OA using ultrasound [24]. A recent systematic review of ultrasound in OA by Keen [14] and a review by Iagnocco [7] stated the need for further work to validate ultrasound features of OA.

There have been no previous studies that have assessed the reliability and validity of ultrasound features of OA in a community cohort of elderly subjects. The objective of this study was therefore to measure the inter-rater reliability and validity of ultrasound imaging in the detection of various features of OA in a community cohort of the elderly.

## Methods

### Study population

Forty surviving participants from the Northumberland Over 85 cohort were invited to participate in this study by sending a request letter in the post. This is a prospective community study of subjects aged 85 years or older from one General Practice in Northumberland, UK, that commenced in 2006. Twenty participants volunteered to participate and were invited to attend the Alnwick Infirmary in August 2009, to undergo weight bearing antero-posterior radiographs of both knees and also had ultrasound assessment of both knees for features of OA, by a trained ultrasonographer (AA). Two replaced joints were not imaged.

**Figure 1:**
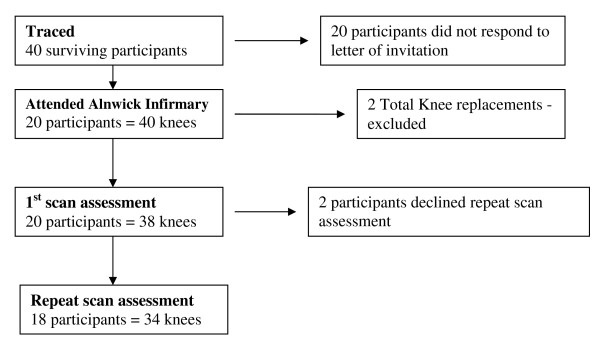
**Flow chart to show numbers of knees scanned for inter-rater reliability at the first and the repeat scan assessments**.

18 of these participants (34 knees) were re-examined using ultrasound up to six weeks later for the same features by a different trained sonographer in September 2009 (Figure 1). Two participants declined the repeat scan. Five subjects opted to have the repeat scan at home.

A local ethics committee (Northumberland Research Ethics Committee, Tyne and Wear, UK) approved the study, which fulfilled the requirements of the Declaration of Helsinki of 1975, as revised in 2000 and the procedures followed were in accordance with the ethical standards of the committee on human experimentation. Written informed consent was obtained from all participating individuals.

### Ultrasound assessment

All ultrasound assessments were performed using the same machine with a 10-18 MHz linear transducer (Mylab 5; ESAOTE, Genoa, Italy). Both sonographers had spent time together, comparing their acquisition and reading techniques on a separate cohort of patients, to arrive at a consensus prior to the commencement of this study.

**Figure 2:**
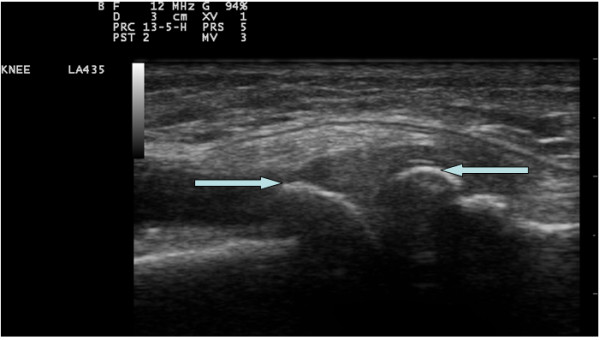
**Femoral (left arrow) and tibial (right arrow) osteophytes - medial longitudinal view**.

**Figure 3:**
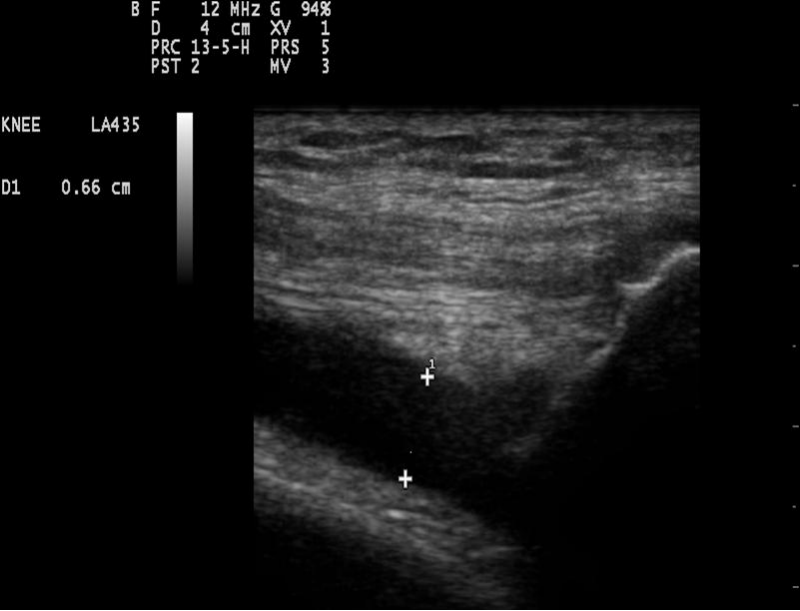
**Knee effusion in longitudinal supra-patellar view (hypoechoic area between the two markers)**.

**Figure 4:**
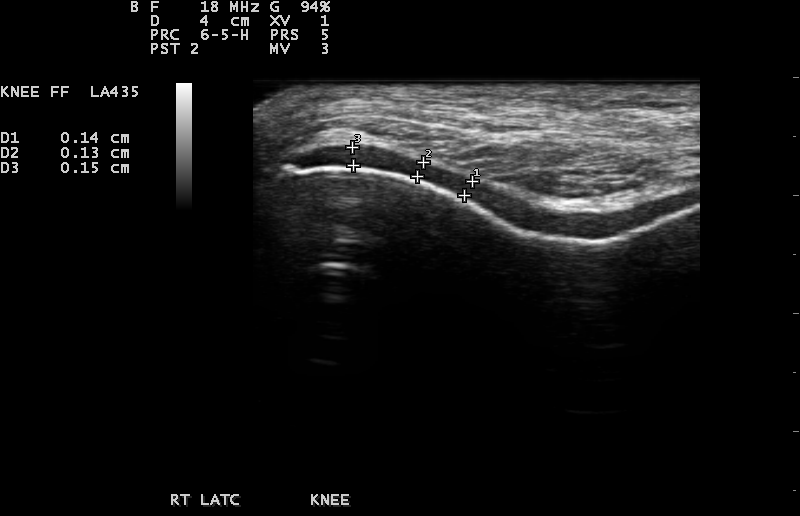
**Femoral cartilage thickness at right lateral condyle (three separate measurements)**.

The scans were based on a protocol derived from EULAR guidelines [25] while the OMERACT guidelines for synovial effusion [26] were also met. The presence or absence of osteophytes was assessed at the tibial and femoral sites in both knees, with 30 degrees of knee flexion (Figure 2). Osteophytes were defined as cortical protrusions at the joint margin seen in two planes [27]. Femoral and tibial osteophytes were assessed in the medial and lateral compartments using medial and lateral longitudinal scan positions, respectively. 30 degrees flexion of the knees was standardised by using the same wedge for all ultrasound assessments. Synovial effusion was defined as an abnormal anechoic or hypoechoic area in the joint that is displaceable and compressible and lacks Doppler signal; as per the OMERACT guidelines [26]. The size of effusions was measured in the longitudinal supra-patellar position, with the knee in 30 degrees of flexion. The maximum diameter of the effusion in the longitudinal view was used to quantify it (Figure 3). Joint effusion was defined by using a cut off of ≥4 mm effusion depth, as seen in a previous multi-centre European study [28]. The thickness of the femoral condylar cartilage was measured in the medial and lateral condyles and in the notch, with the knee in maximum flexion (Figure 4). Cartilage thickness was measured from the thin hyper-echoic line at the soft tissue-cartilage interface to the hyper-echoic line at the cartilage-bone interface. The probe was placed transversely to the leg and perpendicular to the bone surface, just above the superior margin of the patella; this technique being derived from the methods described by Aisen [29] and Iagnocco [30]. There were no other lines drawn to demarcate the probe position.

### Radiographs

All radiographs were read consecutively by a single trained observer (IG), using the Kellgren and Lawrence (K-L) criteria (0-4, 0 = none, 4 = severe) [1]. Grading included the presence of osteophytes and minimal joint space [31] in the medial and lateral tibio-femoral compartments of the knees. Only definite osteophytes were classified as present, with absent or possible osteophytes classified as not present. The images from all 20 participants (38 knees) were used for comparison between ultrasound and radiographs.

### Statistical analysis

#### Reliability

The thickness at the medial and lateral condyles was measured by taking the mean of three measurements at each site. ICCs were calculated to assess the agreement between observers on the size of effusions and the thickness of the femoral condylar cartilage in the notch, medial and lateral sites. Kappa statistics (κ ) [32] and corresponding 95% confidence intervals (95% CI) were calculated for the agreement between observers on the presence or absence of osteophytes at each site.

#### Validity

Unweighted κ was calculated for the agreement between US and radiographs on osteophytes.

All statistical analyses were performed using the statistical software package Stata, version 10 (StataCorp, College Station:TX).

## Results

The median age of the 20 participants in this study was 89.5 years (88-99 years); 60% (n = 12) were female. For right femoral osteophytes, sonographer 1 (AA) had 37% prevalence while sonographer 2 (GM) had 53%. On the left, AA had 47% and GM had 53% prevalence of femoral osteophytes. AA had 32% right tibial osteophytes while GM had 41%. On the left, AA had 42% and GM had 35%.

The prevalence of effusions (≥4 mm) is as follows: AA had 53% prevalence on the right, while GM had 47%. On the left, AA had 47% and GM had 41%.

The prevalence of radiographic abnormalities defined by K-L criteria is as follows: K-L 1 = 17.5%, K-L 2 = 24%, K-L 3 = 41%, K-L 4 = 17.5%. Definite radiographic osteophytes were present in 44% of subjects.

The results of the inter-rater reliability between the two ultrasound observers and that of the validity of ultrasound imaging when compared to radiographs, are stated below.

### Reliability

κ for osteophyte presence was in the range of 0.65 to 0.88 (Table 1). ICCs for effusion size were 0.70 (right) and 0.85 (left). Similar high kappa values for presence/absence of effusion were found; 0.65 (right) and 0.77 (left). Moderate to substantial agreement was found in cartilage thickness measurements except for the lateral femoral cartilage thickness on the right, which had a raw κ value of 0.06. However, after the exclusion of two outlying values, that had been prospectively flagged (prior to analysis) as being particularly difficult to read by the more experienced ultrasonographer, the ICC for this region was 0.67.

**Table 1:** (Inter-rater reliability: results of comparison between two ultrasound observers)

	Kappa (95% CI)	ICC (95% CI)
Osteophyte (right femur)	0.77 (0.31,1.23)	

Osteophyte (left femur)	0.65 (0.41,1.35)	

Osteophyte (right tibia)	0.88 (0.18,1.12)	

Osteophyte (left tibia)	0.88 (0.41,1.35)	

Effusion size (right)		0.70 (0.45,0.95)

Effusion size (left)		0.85 (0.72,0.98)

Effusion presence (right)	0.65 (0.21, 1.1)	

Effusion presence (left)	0.77 (0.31, 1.23)	

Notch thickness (right)		0.68 (0.43,0.94)

Notch thickness (left)		0.62 (0.32,0.92)

Lateral femoral cartilage thickness (right)		0.06 (0.00,0.54) *

Lateral femoral cartilage thickness (left)		0.50 (0.14,0.86)

Medial femoral cartilage thickness (right)		0.57 (0.25,0.90)

Medial femoral cartilage thickness (left)		0.42 (0.02,0.82)

*after removing the 2 identified outliers, ICC was 0.67 (0.38,0.95)

### Validity

For osteophytes, κ was moderate to substantial at 0.52 (95% CI 0.06, 0.98) (right) and 0.75 (95% CI 0.28, 1.22) (left) when comparing radiograph results with those of the first sonographer (AA). When the results of the second sonographer (GM) were used in the comparison, the corresponding kappa values were 0.45 (95% CI -0.04, 0.94) on the right and 0.57 (95% CI 0.05, 1.09) on the left; demonstrating moderate agreement.

## Discussion

This study shows substantial to excellent agreement between two ultrasound observers for presence of osteophytes and effusion size. Moderate to substantial agreement was also demonstrated for measurement of ultrasound derived femoral cartilage thickness. Moderate to substantial validity was demonstrated when comparing osteophytes detected by ultrasound to those seen on radiographs.

There were some important methodological considerations to note in this study. Assessment of the whole process of both acquisition and reading of ultrasound images was performed: thereby including the main potential sources of variation. Some previous studies have only looked at the reliability in reading images between observers [16], but it is important to measure the differences in the acquisition of images, especially considering the dynamic nature of US imaging. The results of this study are therefore likely to be closer to the true value. Intra-rater reliability was not measured in this study but is likely to be as good as, if not better than, inter-rater reliability.

There was a time interval of up to six weeks between the two ultrasound observations, which might have altered the magnitude (size) of effusions. However, the participants were recruited from the community and not from attendance at either primary or secondary care. Therefore, it is unlikely that they had any significant steroid or other specific therapy in hospital for the incidental effusions that were picked up on the first ultrasound. As effusion size within participants might still have changed during this period, this interval could only have served to decrease the agreement between the two sonographers. The inter-rater agreement for size of effusions found in this study therefore is also likely to be conservative. When effusion was considered as a binary variable (using a cut off of ≥4 mm depth), the κ was 0.65 (right) and 0.77 (left); which remains very close to the ICC values obtained when effusion was used as a continuous variable.

Power Doppler assessment of synovitis (PDS) was not conducted in this study as the machine used for the study did not appear to have adequate sensitivity, based on images acquired prior to the study. PDS has been found to be a valid [33] method of detection of synovitis in the knees, although its reliability is still to be established. A EULAR group that assessed ultrasound features of inflammation decided not to evaluate PDS due to their concern that this was highly machine dependant [28].

This study did not seek to confirm that the osteophytes seen on ultrasound were the same ones on the radiographs, as the presence of any bone response is likely to be clinically important. The kappa values for validity were comparable when either sonographer's osteophyte results were compared with radiographic osteophytes. The confidence intervals of these values between the sonographers overlap significantly; which is reassuring. Previous methods evaluating femoral condylar cartilage [9,10], have used semi-quantitative scores to assess the clarity and sharpness of cartilage, but this has the disadvantage of losing precision due its ordinal scale. In addition, the features of sharpness and clarity are quite likely to differ between the subjective assessments of observers. These features are also susceptible to change as more advanced ultrasound machines with better resolution are created.

The two sonographers agreed on a consensus for the acquisition and reading of images, prior to the commencement of this study. This would have decreased the learning curve that otherwise might have been seen. However, the scanning protocols did not include restrictive methods such as the use of grid lines to assist the placement of the probe for cartilage thickness measurement, as has been seen with previous studies [29,30]. It is important that sonographers refer to the guidelines suggested by Backhaus et al [25] so that consistency can be achieved in future studies using ultrasound as an outcome measure in OA.

The demonstration of reliability and validity is an important precursor to any epidemiological study of OA using ultrasound. Previous studies that have assessed inter-rater reliability of ultrasound features of knee OA have included only small numbers of patients [17] or a small subset of the patients in the original study. Inter-rater reliability between multiple experts in Europe on six patients (two with Rheumatoid Arthritis, four with OA) showed an overall Kappa of 0.60 for the knee, with the agreement being 92% for effusion/synovitis and 85% for bony cortex abnormalities [17]. Our study results show a slight improvement in the agreement between two ultrasound observers, when compared to a study of the knee [15] and the hip [16] previously. This may in part be due to the fact that the two observers in our study had the opportunity to agree on a consensus, prior to the commencement of the study. This is the largest study to date, involving 34 knees, to address the issue of inter-rater reliability of ultrasound for various features of knee OA.

Jonsson et al [34] studied six patients and four controls who had each of these imaging modalities repeated once within one to four weeks of the initial imaging procedure. Radiographs (although an indirect measure of cartilage thickness) were the most reproducible imaging modality to assess cartilage in the knees with a co-efficient of variation of 6.5%, while ultrasound performed next best with a co-efficient of variation of 8.4% and magnetic resonance imaging faring worst at 12%. While this may suggest that ultrasound demonstrates better test-retest reliability than MRI, it should also be noted that significant improvements have been made in the quantification of cartilage measurements by MRI [35] since that study took place. The kappa and ICC agreement values in our study using ultrasound are comparable to those of radiographic studies of inter-rater reliability [36,37].

Ultrasound demonstrated excellent agreement with MRI in a validation exercise involving 14 observers from all around Europe. There was 100% agreement for effusion, 79% for synovial hypertrophy and 75% agreement for osteophytes, when compared to MR imaging, among the observers who imaged the knees of four patients with inflammatory arthritis [18]. Yoon et al demonstrated validity of the longitudinal sagittal ultrasound image for assessment of cartilage thickness in a study using MRI as the comparator in 51 patients with knee OA in South Korea [38]. However, the longitudinal sagittal image has not been performed subsequently in other studies or advocated previously in the EULAR guidelines [25]; this was not performed in our study either. The transverse image for femoral cartilage thickness has been validated by comparison with histopathological specimens [19], which can be considered to be the gold standard and superior to MRI in measurement of cartilage thickness. Naredo et al compared ultrasound with measures of pain and radiographs in 50 patients with knee OA [39]. They showed that knee effusion, medial meniscal protrusion and displacement of the medial collateral ligament were associated with significantly higher knee pain. Medial meniscal protrusion was related to decreased medial joint space width on radiographs. A Danish study which compared ultrasound and MRI showed that ultrasound detected 100% of effusions seen on MRI and Spearman coefficients of 0.87 and 0.86 were seen for effusion and synovial thickness measurements between the two modalities, respectively [22]. Our study could not validate features of inflammation because the comparator was radiographs. However, the Kappa values of 0.52 and 0.75 for comparison of osteophyte detection between ultrasound and radiographs in our study are similar to the results from the MRI study above.

## Conclusions

In summary, there has been a concern that ultrasound is a very "operator dependant" imaging modality; this study provides evidence that high inter-rater reliability can be achieved between observers for the features of osteophytes, synovial effusion and femoral cartilage thickness. Osteophytes have been known to be an integral part of the pathophysiology of OA and shown in some studies to be significantly associated with pain in the knee [2,40], although other studies [41] have been unable to find this association.. Ultrasound is also valid for features such as osteophytes, especially at the knee.

This is the first study to look at inter-rater reliability and validity of ultrasound features of OA in the community, where ultrasound is a reliable tool. More evidence is needed for its validity in this setting. Future studies should look at the construct validity of ultrasound by comparing against MRI and symptoms of OA. Also, the predictive validity of ultrasound should be measured in longitudinal community cohort studies.

## List of abbreviations

OA: Osteoarthritis; MRI: Magnetic resonance imaging; K: Kappa; ICC: Intra-class correlation co-efficients

## Competing interests

The authors declare that they have no competing interests.

## Authors' contributions

AMA was involved in the design of the study, collection of data; acquisition and reading of ultrasound images; analysis and interpretation of data and drafting of the article. IG read the radiographs, helped with the interpretation of data and with critical revision of article for important intellectual content. MSP participated in the design of the study, provided statistical expertise and helped with the critical revision of article for important intellectual content. RMF participated in the conception and design of the study and in the critical revision of article for important intellectual content. FB conceived and designed the study, obtained funding and helped with the interpretation of data and critical revision of article for important intellectual content. All authors read and approved the final manuscript.

## Pre-publication history

The pre-publication history for this paper can be accessed here:

http://www.biomedcentral.com/1471-2474/12/70/prepub
